# IL-17A deficiency inhibits lung cancer-induced osteoclastogenesis by promoting apoptosis of osteoclast precursor cells

**DOI:** 10.1371/journal.pone.0299028

**Published:** 2024-02-23

**Authors:** Hongkai Wang, Hao Tang, Shujie Yuan, Chuntao Liang, Yuanxin Li, Shida Zhu, Kai Chen

**Affiliations:** 1 Department of Orthopedics, The Second Affiliated Hospital of Guilin Medical University, Guilin, Guangxi, China; 2 Guangxi Key Laboratory of Metabolic Reprogramming and Intelligent Medical Engineering for Chronic Diseases, The Second Affiliated Hospital of Guilin Medical University, Guilin, Guangxi, China; Universite de Nantes, FRANCE

## Abstract

Osteoclasts are crucial in the events leading to bone metastasis of lung cancer. Interleukin-17A (IL-17A) affects osteogenesis by regulating the survival of osteoclast precursors (OCPs) and is enriched in lung cancer cells. However, how factors derived from tumor cells that metastasize to bone affect osteoclastogenesis remains poorly understood. We examined whether IL-17A derived from lung cancer cells affects osteoclast differentiation by regulating OCP apoptosis. IL-17A expression was inhibited in A549 non-small cell lung cancer cells using RNA interference. Compared with conditioned medium (CM) from A549 cells (A549-CM), CM from IL-17A-deficient A549 cells (A549-si-CM) suppressed osteoclastogenesis. The mRNA expression of osteoclast-specific genes was downregulated following A549-si-CM treatment. Furthermore, A549-si-CM promoted osteoclast precursor apoptosis at an early stage of osteoclastogenesis, which was related to the promotion of caspase-3 expression by A549-si-CM during osteoclast differentiation. *In vivo* experiments also showed that inhibition of IL-17A expression in A549 cells reduced osteoclast activation and bone tissue destruction. Collectively, our results indicate that IL-17A deficiency inhibits lung cancer-induced osteoclast differentiation by promoting apoptosis of osteoclast precursors in the early stage of osteoclast formation and that IL-17A is a potential therapeutic target for cancer-associated bone resorption in patients with lung cancer.

## Introduction

Lung cancer is a malignant tumor with a high incidence and mortality rate worldwide [1]. Among lung cancer cases, 80–85% are non-small cell lung cancer (NSCLC) [2]. Although diagnostic methods for lung cancer are improving, distant metastasis often occurs [3], preventing patients from undergoing radical surgery and leading to various complications [4]. Bone tissue is a common site of distant metastasis. Bone metastasis (BM) is observed in approximately 30–40% of patients with advanced NSCLC [5]. Bone metastasis of lung cancer directly or indirectly activates osteoclasts, which further accelerates tumor–BM while destroying bone tissue. These effects result in complications such as hypercalcemia and pathological fractures [4]. Thus, osteoclast activation plays an important role in the process of BM in lung cancer [6]. However, how factors derived from tumor cells that metastasize to bone affect osteoclastogenesis remains poorly understood.

IL-17A is the main bioactive member of the interleukin (IL)-17 family [7]. Previous studies reported high IL-17A expression in lung cancer cells and in the serum of patients with NSCLC that was associated with poor prognosis [8]. In addition, many studies showed that IL-17A affects bone remodeling [9, 10]. Exogenous IL-17A affects autophagy in osteoclast precursors (OCPs), thus affecting osteoclast differentiation [11]. Apoptosis refers to programmed cell death that occurs during development or under the influence of specific factors [12]. IL-17A affects osteoclast differentiation by regulating OCP apoptosis [13]. However, it is unclear whether IL-17A derived from lung cancer cells affects osteoclastogenesis by regulating OCP apoptosis.

Considering the established role of IL-17A in lung cancer development, we investigated the effects of IL-17A derived from lung cancer A549 cells on osteoclast differentiation.

## Material and methods

### Cell culture

A549 human lung adenocarcinoma epithelial cells and RAW 264.7 murine macrophages were obtained from American Type Culture Collection (Manassas, VA, USA). The cells were cultured as described in our previous study [14].

### RNA interference

Small interfering RNA (siRNA) targeting human IL-17A was synthesized by OBiO Technology (Shanghai, China). The target sequences were as follows: 5′-CCTAAGGTTAAGTCGCCCTCG-3′ (snc) and 5′-CCCAAATTCTGAGGACAAGAA-3′ (si). siRNAs were transfected into A549 cells using Lipofectamine 3000 reagent (Thermo Fisher Scientific, Waltham, MA, USA), following the manufacturer’s instructions [11].

### Preparation of conditioned medium

To prepare conditioned medium (CM), A549, A549-snc, and A549-si cells were seeded into 100-mm dishes and cultured until 70% confluency. The medium was then replaced with serum-free medium. After 48 h, the media was collected and centrifuged (1000 ×*g* for 10 min), and the supernatant was collected and stored at −80°C until use as the CM. To adjust for varying cell densities due to proliferation, the cell count and CM volume were normalized across samples [14]. The CM from A549 cells transfected with A549-si (A549-si-CM), A549-snc (A549-snc-CM), or untransfected cells (A549-CM) was diluted 1:1 in Dulbecco’s Modified Eagle Medium and used for RAW 264.7 cell culture in the presence of receptor activator of nuclear factor κB ligand (RANKL) (50 ng/ml, R&D Systems, Minneapolis, MN, USA).

### Tartrate-resistant acid phosphatase staining

Multinucleated osteoclast-like cells were stained for tartrate-resistant acid phosphatase (TRAP) using a TRAP/ALP Stain Kit (FUJIFILM, Tokyo, Japan) according to the manufacturer’s instructions. Multinucleated TRAP-positive cells with three or more nuclei were considered as osteoclasts.

### RNA isolation and real-time quantitative PCR

Total RNA was isolated from cells using TRIzol Reagent (Beyotime, Wuhan, China) according to the manufacturer’s instructions. Equal amounts of total RNA from each sample were reverse-transcribed into cDNA using the PrimeScript RT Reagent Kit with cDNA Eraser (Takara, Shiga, Japan). Real-time quantitative PCR was performed as previously described [11]. Primer sequences are listed in S1 Table.

### Analysis of apoptosis

After treatment with A549-CM or A549-si-CM, the RAW264.7 cells were collected and suspended in phosphate-buffered saline (PBS). The cells were suspended in binding buffer, stained using an Annexin V-FITC/PI apoptosis detection kit (Beyotime), and evaluated using a BD Accuri C6 flow cytometer (BD Biosciences, Franklin Lakes, NJ, USA) [11].

### Western blot analysis

RAW264.7 cells were cultured for 24 h in A549-CM or A549-si-CM in the presence of RANKL (50 ng/ml). Total proteins were extracted using radioimmunoprecipitation assay buffer (Beyotime). Western blot analysis was performed as previously described [11]. The primary monoclonal antibody against IL-17A (cat No. 66148, 1 μg/mL) was obtained from Proteintech (Rosemont, IL, USA). Primary monoclonal antibodies against Bcl2 (cat No.3498, 0.34 μg/mL), p53 (cat No. 2524, 0.31 μg/ml), cleaved caspase-3 (CASP3, cat No. 9664, 0.04 μg/mL), caspase-9 (CASP9, cat No. 9508, 0.83 μg/mL), β-actin (cat No. 4970, 0.06 μg/mL), and primary polyclonal antibody against BAX (cat:2772, 0.11 μg/mL), were obtained from Cell Signaling Technology (Danvers, MA, USA). We obtained HRP conjugated goat anti-rabbit IgG (cat No. BA1050, 0.2 μg/mL) and HRP conjugated goat anti-mouse IgG (cat No. BA1054, 0.2 μg/mL) secondary antibodies from Boster Bio (Wuhan, China).

### Animal model of tumor bone metastasis

This study was performed in strict accordance with the recommendations in the Guide for the Care and Use of Laboratory Animals of the National Institutes of Health. The protocol was approved by the Committee on the Ethics of Animal Experiments of Guilin Medical University (Protocol Number: GLMC-IACUC-2023002). Special training in animal handling was provided for research staff. All surgery was performed under sodium pentobarbital anesthesia, and all efforts were made to minimize suffering. Twenty male six-week-old immunodeficient BALB/c-nu/nu mice were obtained from Hunan SJA Laboratory Animal Co., Ltd. (Changsha, China) and maintained under sterile conditions. All animals were housed with water and food available *ad libitum* in temperature- and humidity-controlled rooms (22 ± 1°C and 60% humidity) with a 12-h light/dark cycle throughout the experimental period. A549 and A549-si cells were cultured and resuspended in PBS at a final concentration of 10^6^ cells/mL. Mice were anesthetized by intraperitoneal injection of pentobarbital (50 mg/kg). A syringe with a 26 1/2 G needle was inserted in the proximal end of the tibia to inject tumor cells (10^4^ cells in 10 μL PBS) into the intramedullary space [15]. To monitor animal health, the body weight of mice was recorded every 2 days.

### Assessment of osteolytic lesions *in vivo*

No animals died before euthanasia was administered. To further characterize osteolytic bone lesions caused by BM after injection of tumor cells into the tibia for 4 weeks, the mice were euthanized via cervical dislocation after being anesthetized with sodium pentobarbital (150 mg/kg). We then performed X-ray imaging on the mice, using the IVIS Lumina XRMS Series III In Vivo Imaging Device at 35 kVp for 2 min (PerkinElmer, Waltham, MA, USA). Mice were imaged and evaluated in a blinded manner. To further observe osteoclast activation, the tibias were dissected and fixed in 4% paraformaldehyde (Beyotime) for 48 h. The tissue samples were decalcified in EDTA and embedded in paraffin. Sections were cut for hematoxylin-eosin (HE) staining and TRAP staining as previously described [15]. The area of osteolytic destruction was quantified by manually selecting the osteolytic areas using ImageJ software (version 1.8.0; NIH, Bethesda, MD, USA) [16].

### Statistical analysis

All experiments were performed in at least triplicate. Significant differences between two groups were determined using Student’s *t*-test and between multiple groups using one-way analysis of variance and Tukey’s test. All statistical analysis was performed using SPSS Statistics software (version 20.0; SPSS, Inc., Chicago, IL, USA). Data are presented as the mean ± standard deviation. P < 0.05 was considered to indicate statistically significant results.

## Results

### SiRNA-mediated silencing of IL -17A expression in A549 cells

Specific siRNA targeting *IL-17A* mRNA was used for knockdown of IL-17A expression in A549 cells, which was confirmed using real-time quantitative PCR and western blotting. A549-si suppressed *IL-17A* mRNA and protein expression compared to that in control-transfected cells (S1 Fig).

### CM from IL-17A-deficient A549 cells inhibited RANKL-stimulated osteoclastogenesis

**Fig 1:**
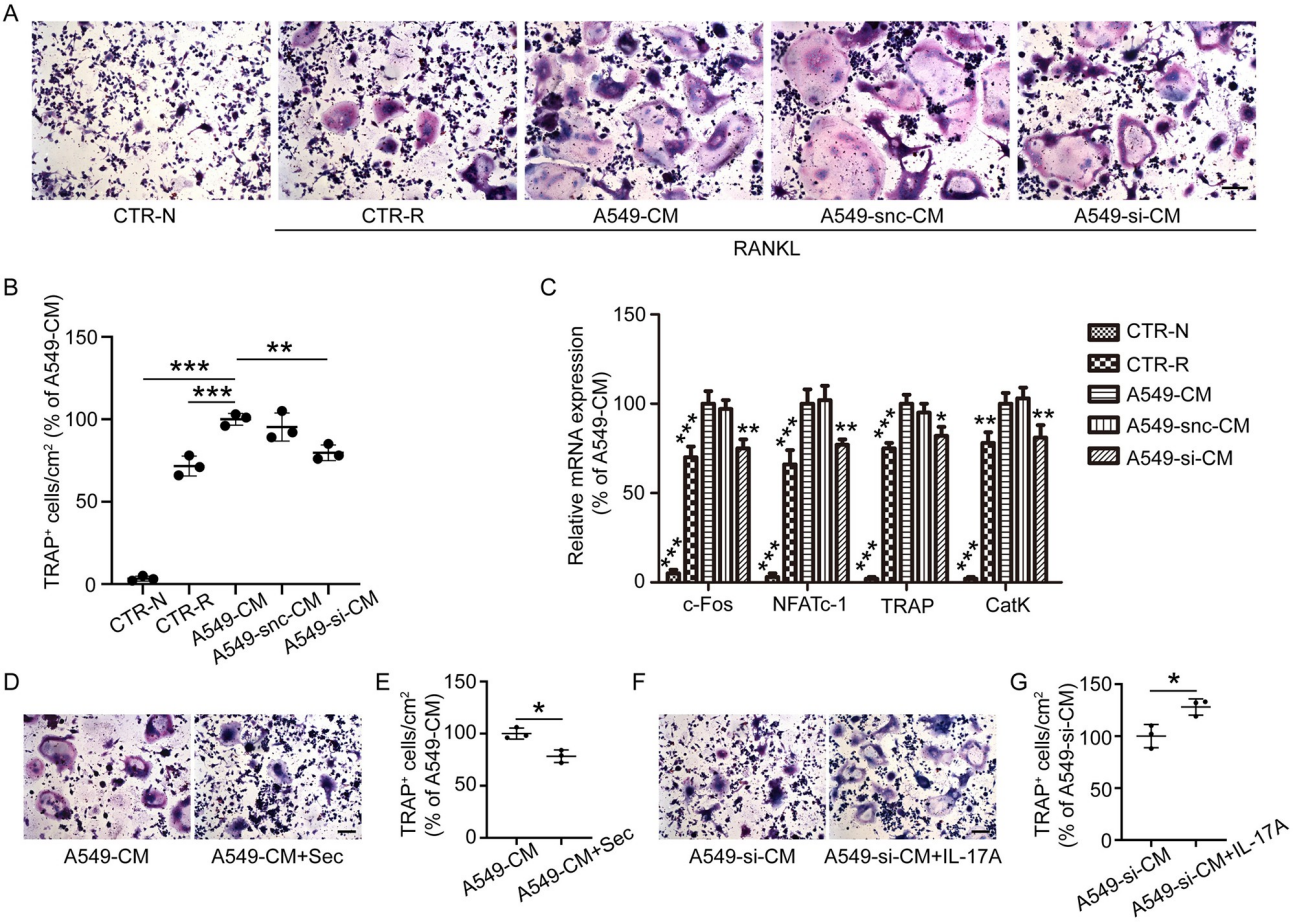
Conditioned medium (CM) from IL-17A-depleted A549 cells suppresses RANKL-induced osteoclastogenesis and the expression of osteoclast-related genes. (A) RAW 264.7 cells were cultured with CM from A549 cells transfected with siRNA against IL-17A (A549-si-CM), a negative control (A549-snc-CM), or from untransfected cells (A549-CM) in the presence of RANKL for 5 days. (B) Quantitative analysis of tartrate-resistant acid phosphatase (TRAP)-positive multinucleated (≥3 nuclei) RAW264.7 cells cultured in CM. (C) Transcript levels of *c-Fos*, *NFATC-1*, *TRAP*, and *CatK* in cells treated with A549-si-CM, A549-snc-CM, or A549-CM in the presence of RANKL for 48 h. Data represent the fold-changes in target gene expression normalized to that of glyceraldehyde-3-phosphate dehydrogenase (*GAPDH*) and are expressed as a percentage of the levels in cells treated with A549-CM, which was set to 100%. (D) RAW 264.7 cells were cultured in A549-CM with or without secukinumab (an IL-17A monoclonal antibody) in the presence of RANKL for 5 days. (E) Quantitative analysis of TRAP-positive multinucleated (≥3 nuclei) RAW264.7 cells cultured in A549-CM with or without secukinumab. (F) RAW 264.7 cells were cultured in A549-si-CM with or without IL-17A (1 ng/mL) in the presence of RANKL for 5 days. (G) Quantitative analysis of TRAP-positive multinucleated (≥3 nuclei) RAW264.7 cells cultured in A549-si-CM with or without IL-17A. Values represent the mean ± standard deviation (SD) of experiments performed independently in triplicate. CTR-N, control group treated without RANKL; CTR-R, control group treated with RANKL. *P < 0.05, **p < 0.01, ***p < 0.001. Scale bar, 100 μm.

To determine whether IL-17A silencing affects osteoclast formation, A549-CM, A549-si-CM, or A549-snc-CM was diluted 1:1 in Dulbecco’s Modified Eagle Medium and added to RAW 264.7 cell cultures in the presence of RANKL for 5 days. RAW 264.7 cells exposed to RANKL showed an increase in TRAP-positive multinucleated osteoclasts; addition of A549-si-CM suppressed this increase compared to that in A549-CM or A549-snc-CM-treated cells (Fig 1A and 1B). To confirm that the depletion of 1L-17A itself was the main and direct cause of impaired osteoclastogenesis, secukinumab (10 μg/ml, an IL-17A monoclonal antibody) was added to A549-CM. The results showed that inhibiting IL-17A alleviated the promoting effect of A549-CM on osteoclastogenesis (Fig 1D and 1E). Moreover, exogenous IL-17A rescued the inhibitory effect of osteoclastogenesis induced by A549-si-CM (Fig 1F and 1G).

### Expression of osteoclast-related genes was downregulated following treatment with CM from IL-17A-deficient A549 cells

RAW 264.7 cells were treated with A549-CM, A549-snc-CM, and A549-si-CM in the presence of RANKL for 48 h, and the expression of osteoclast-related genes was examined. *TRAP* mRNA levels were downregulated in cells treated with A549-si-CM compared to the levels in cells treated with A549-snc-CM or A549-CM. Similar trends were observed for the mRNA levels of *c-Fos*, *NFATC-1*, and *CatK* (Fig 1C).

### CM from IL-17A-depleted A549 cells promoted OCP apoptosis at an early stage of osteoclast differentiation

**Fig 2:**
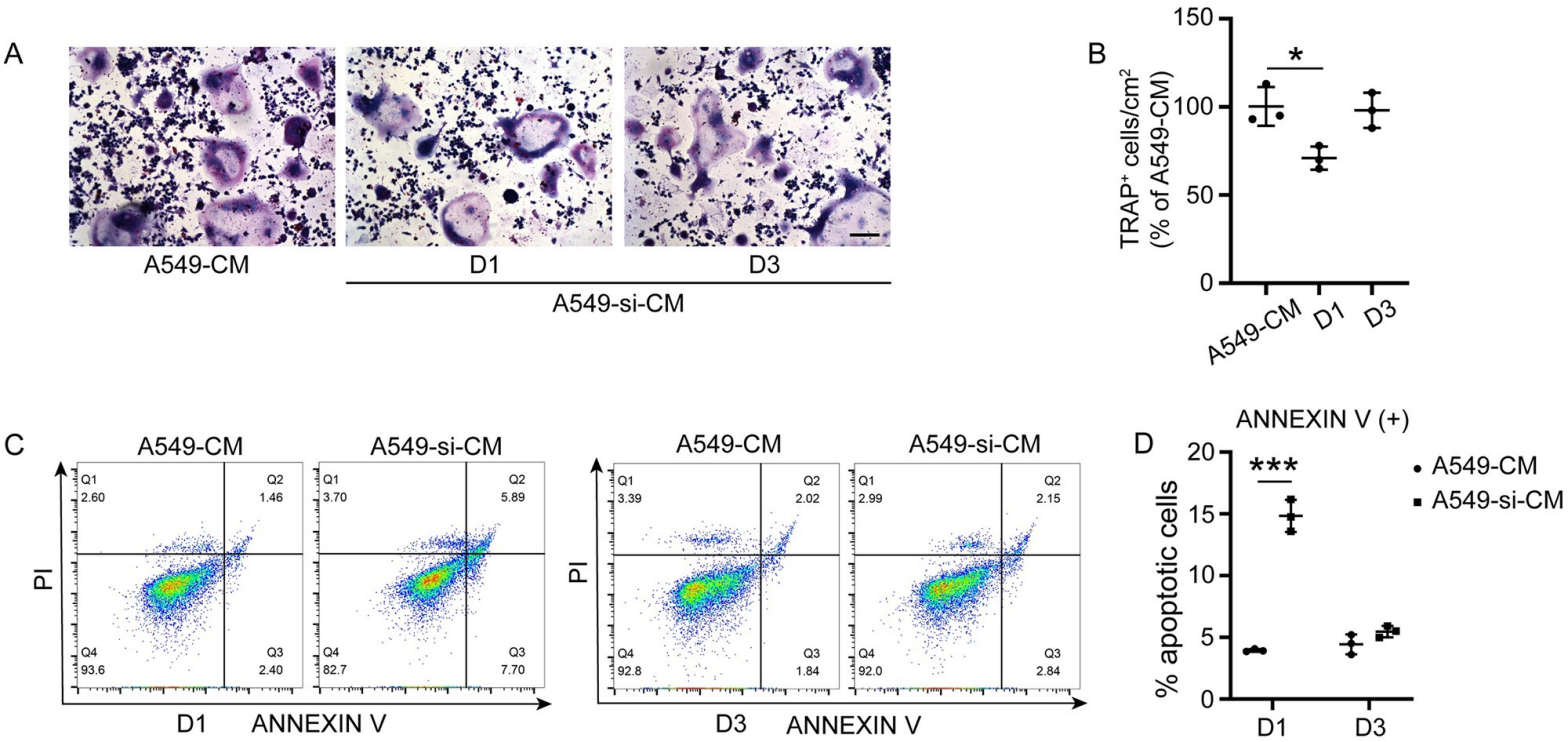
Conditioned medium (CM) from IL-17A-depleted A549 cells promotes osteoclast precursor (OCP) apoptosis at an early stage of osteoclast differentiation. (A) RAW 264.7 cells were cultured in A549-CM or A549-si-CM in the presence of RANKL for 5 days. A549-si-CM was added to the culture medium on the first (D1) and third days (D3), respectively. (B) Quantitative analysis of TRAP-positive multinucleated (≥3 nuclei) RAW264.7 cells. (C) Annexin V-FITC/PI staining was performed to label apoptotic cells. The percentages of apoptotic cells (Annexin V+) were counted using flow cytometry, and (D) Q2 and Q3 quadrants indicated increased apoptosis after A549-si-CM treatment on the first day. Cells treated with A549-CM were used as controls. Data are presented as the mean ± SD from three independent experiments. *p < 0.05, ***p<0.001. Scale bar, 100 μm.

To identify the stage at which A549-si-CM inhibits osteoclast differentiation, A549-si-CM was added at different time points. Addition of A549-si-CM on the first day considerably inhibited osteoclast differentiation, whereas addition of A549-si-CM on the third day had no inhibitory effect (Fig 2A and 2B). Flow cytometry was performed to verify the effect of A549-si-CM on OCP apoptosis at an early stage of osteoclast differentiation. At this stage, there was a considerable increase in the number of annexin V-positive cells in RAW264.7 cells treated with A549-si-CM compared to that of those treated with A549-CM (Fig 2C and 2D).

### CM from IL-17A-depleted A549 cells inhibited osteoclast differentiation by promoting CASP3 expression

**Fig 3:**
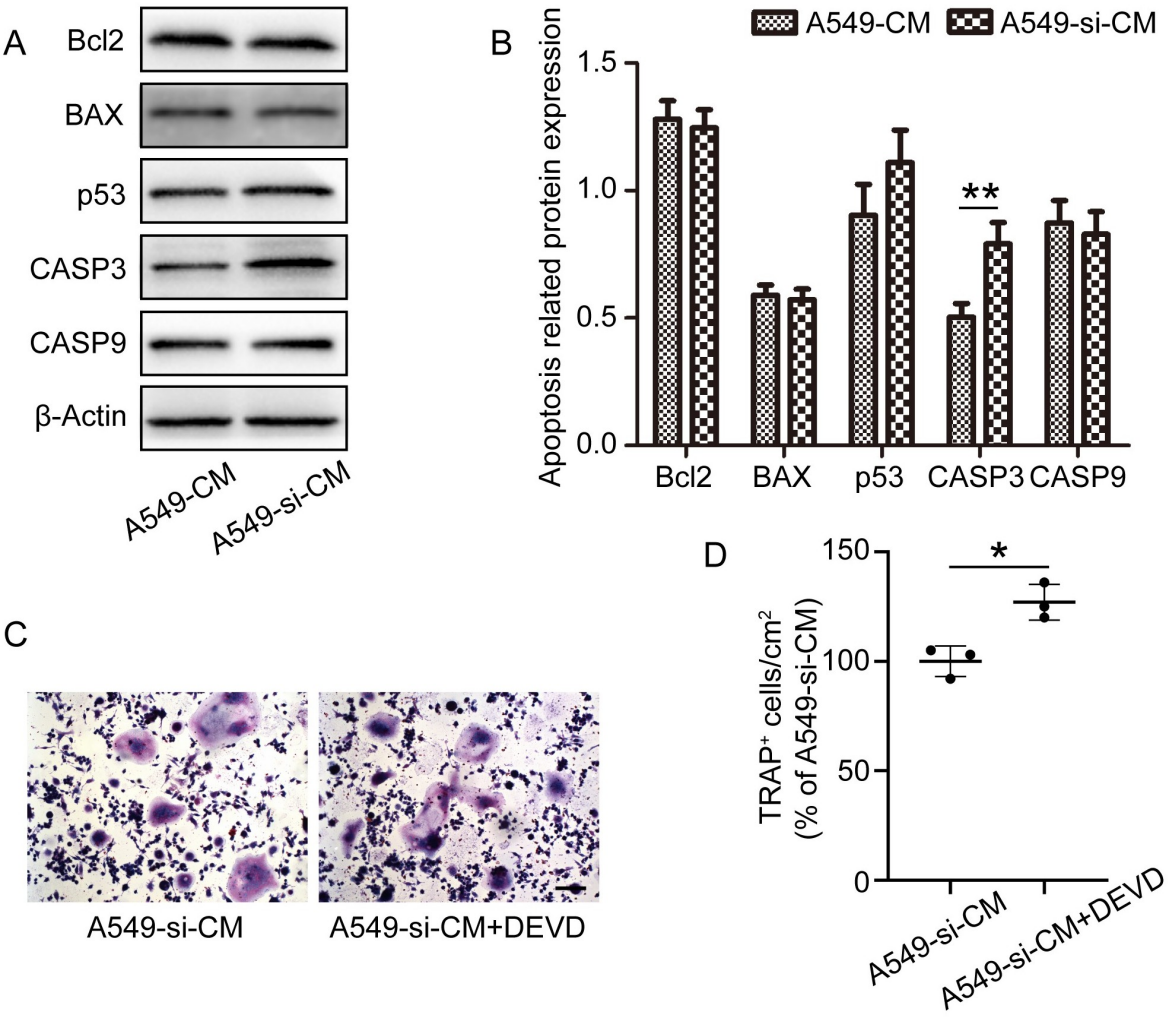
Conditioned medium (CM) from IL-17A-depleted A549 cells inhibits osteoclast differentiation by promoting CASP3 expression. (A) RAW 264.7 cells were cultured in A549-CM or A549-si-CM in the presence of RANKL for 24 h. (A) Apoptosis-related proteins were detected via western blotting, and (B) protein expression was normalized against β-actin. (C) RAW 264.7 cells were cultured in A549-si-CM with or without Z-DEVD-FMK (a CASP3 inhibitor) in the presence of RANKL for 5 days. (D) Quantitative analysis of TRAP-positive multinucleated (≥3 nuclei) RAW264.7 cells. Data represent the mean ± SD of experiments performed independently in triplicate. *p < 0.05, **p < 0.01. Scale bar, 100 μm.

After treatment with A549-CM and A549-si-CM for 24 h, the mRNA expression of the apoptosis markers *Bcl2*, *BAX*, *p53*, *CASP3*, and *CASP9* in RAW264.7 cells was assessed using quantitative PCR. *CASP3* expression was increased in cells treated with A549-si-CM compared to that in cells treated with A549-CM. However, changes in *Bcl2*, *BAX*, *p53*, and *CASP9* mRNA expression were not significant (S2 Fig). In addition, western blotting results showed that CM from IL-17A-depleted A549 cells promoted CASP3 expression (Fig 3A and 3B). Moreover, the inhibitory effect of A549-si-CM on osteoclast differentiation was reversed by treatment with Z-DEVD-FMK, a CASP3 inhibitor (Fig 3C and 3D).

### Silencing of IL-17A expression in A549 cells inhibited osteoclastogenesis *in vivo*

**Fig 4:**
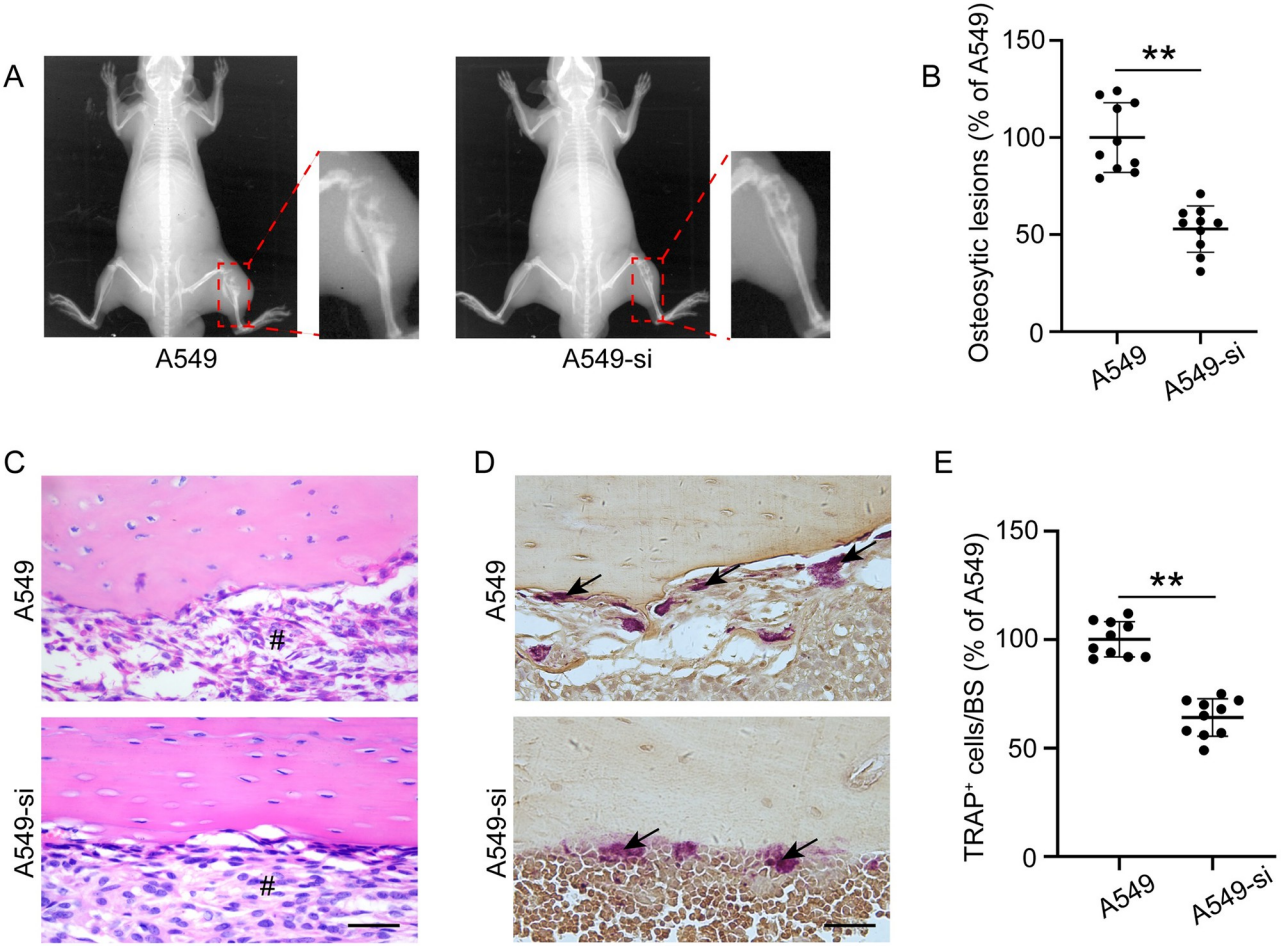
IL-17A-depleted A549 cells prevent tumor metastasis-induced bone destruction *in vivo*. (A) X-ray images of the tibias of mice at 4 weeks after A549 cell-injection. (B) Quantitative analysis of osteolytic bone destruction. (C) Hematoxylin and eosin (HE) and (D) TRAP staining of one representative tibia from each group. (E) Quantitative analysis of osteoclast numbers in bone metastases of A549 cells. Data are expressed as number of osteoclasts/mm at the tumor-bone interface and the mean ± SD. Arrows indicate osteoclasts; # tumor mass. **p < 0.01. Scale bar, 50 μm.

To identify osteolytic bone lesions, bone tissues were subjected to X-ray analysis. As shown in Fig 4A, osteolytic bone destruction of the cortices occurred in A549 tumor-bearing control mice. Conversely, A549-si treated mice exhibited reduced osteolysis (Fig 4B). Notably, HE staining of the tibial slices revealed a large distribution of tumor cells around osteolytic bone lesions (Fig 4C). Histomorphometric analysis of TRAP staining showed that the number of osteoclasts was considerably reduced in mice treated with A549-si compared to that in mice treated with A549 cells (Fig 4D and 4E). These results indicate that IL-17A derived from A549 cells suppressed tumor-induced osteolytic bone lesions *in vivo* by inhibiting osteoclast differentiation.

## Discussion

Recently, the incidence and mortality rates of lung cancer have increased. Each year, more than 2 million people worldwide are diagnosed with lung cancer, resulting in >1.8 million new deaths, ranking first among all cancer types in terms of mortality [17]. Death of patients with lung cancer is typically caused by recurrence or metastasis. The bone tissue is a common site of hematogenous metastasis [18]. Patients with BM have a 5-year survival rate of <5% [19]. Osteolytic lesions often occur as a result of tumor metastasis to the bone, and osteoclast differentiation is a critical event in tumor-induced bone loss [20]. Our findings suggest that IL-17A derived from lung cancer cells affects osteoclast differentiation by regulating OCP apoptosis, indicating that IL-17A is a potential therapeutic target for cancer-associated bone resorption in patients with lung cancer.

IL-17A is highly expressed in cancer cells and promotes tumorigenesis in NSCLC [21]. Several studies have shown that low concentrations of IL-17A promote osteoclastogenesis, whereas high concentrations of IL-17A inhibit osteoclastogenesis [13, 22]. Other evidence suggests that the ability of IL-17A to promote osteoclast differentiation increases with increasing IL-17A concentrations [23]. Considering the correlation between IL-17A and poor prognosis of patients with tumors and the effect of IL-17A on osteoclastogenesis, we used RAW264.7 cells and an animal model of BM to assess the effect of IL-17A isolated from lung cancer cells on osteoclastogenesis and its underlying mechanisms. Unlike previous studies that mostly used exogenous IL-17A to study its effect on osteoclastogenesis [11, 13], we report the promoting effect of IL-17A derived from A549 cells on osteoclast differentiation.

During osteoclast differentiation, a series of genes is expressed that reflect osteoclast activation [11]. Wang et al. [24] reported that hypoxia-inducible factor 1alpha enhanced RANKL-induced osteoclast differentiation by promoting the expression levels of osteoclast-specific genes. To further verify the promoting effect of IL-17A derived from A549 cells on osteoclastogenesis, the expression of osteoclast activation-related mRNA was assessed. *TRAP* mRNA expression was downregulated in cells treated with A549-si-CM compared to that in cells treated with A549-CM or A549-snc-CM. Similar trends were observed for *c-Fos*, *NFATC-1*, and *CatK*. This result is consistent with the findings of Song et al. [23], who found that the expression of *c-Fos*, *NFATC-1*, *CatK*, and *TRAP* increased upon recombinant IL-17A treatment in mouse bone marrow macrophages. However, Kitami et al. [25] observed that different concentrations of IL-17A inhibit osteoclast differentiation and CatK expression. The reason for the different results may be the different experimental conditions used, such as different sources of IL-17A.

Several studies showed that various factors affect osteoclast differentiation at different stages of osteoclastogenesis. Yang et al. [26] demonstrated that oat seedling extract suppressed osteoclast differentiation in the early stage of differentiation. Ishida et al. [27] suggested that osteocalcin fragments in the bone matrix are involved in osteoclast maturation, particularly during the late stages of osteoclast differentiation. Our results showed that the inhibitory effect of A549-si-CM on osteoclastogenesis occurred in the early stages of osteoclast differentiation. Further analysis confirmed that A549-si-CM treatment led to notable OCP apoptosis when CM was added at an early stage; however, this effect was not significant when CM was added at a late stage. These results indicate that the inhibitory effect of A549-si-CM on osteoclastogenesis is achieved through promotion of OCP apoptosis in the early stages of osteoclast differentiation.

The survival of OCPs plays an important role in osteoclastogenesis [28, 29]. Xue et al. [13] reported that low concentrations of IL-17A recombinant protein reduced the apoptosis of OCPs by inhibiting the expression of CASP3. Studies showed that IL-17A affects apoptosis by regulating the expression of apoptotic proteins, such as Bcl2, BAX, p53, CASP3, and CASP9 [30, 31]. In the present study, we observed an increase in CASP3 expression after A549-si-CM treatment compared with that after A549-CM treatment; however, changes in Bcl2, BAX, p53, and CASP9 expression were not significant. Moreover, CASP3 inhibition by Z-DEVD-FMK, an inhibitor of CASP3, reversed the inhibitory effect of A549-si-CM on osteoclastogenesis. These results suggest that IL-17A-deficient lung cancer cells increase CASP3 expression, which promotes OCP apoptosis and inhibits osteoclast differentiation.

*In vivo* studies have shown that IL-17A promotes tumorigenesis in NSCLC [32]. We found that in mice, downregulation of IL-17A expression in A549 cells prevented bone destruction caused by tumor metastasis *in vivo*, with a significant reduction in the number of osteoclasts on the bone surface in mice injected with IL-17A-deficient A549 cells. These results suggest that IL-17A is a therapeutic target for the treatment of lung cancer-induced bone lesions. However, the animal model of BM used in our study could not fully simulate the entire process of tumor BM observed in patients, which is one limitation of this study.

In summary, we showed that inhibiting IL-17A expression in lung cancer A549 cells inhibited osteoclast differentiation, as IL-17A deficiency induced OCP apoptosis in the early stage. The inhibitory effect of low IL-17A expression in A549 cells on osteoclast differentiation was verified in animal experiments. Considering the promoting effect of IL-17A on the invasion and metastasis of lung cancer, targeted therapy with IL-17A may inhibit both tumorigenesis and bone destruction. Another limitation of this study is that other lung cancer cell lines and bone marrow-derived osteoclasts were not used to validate the findings. Further research on the effects of IL-17A on the survival and bone resorption function of mature osteoclasts may support the potential of IL-17A as a therapeutic target for the prevention and treatment of tumor BM.

## Supporting information

S1 Fig Effects of siRNA-mediated IL-17A silencing in A549 cells. (PDF)

S2 Fig Expression of apoptosis-related mRNA after treatment with A549-CM and A549-si-CM. (PDF)

S1 Table Sequences of primers used in RT-PCR analysis. (PDF)

S1 Raw images (PDF)
