# Supplementary material for: IL-17A deficiency inhibits lung cancer-induced osteoclastogenesis by promoting apoptosis of osteoclast precursor cells
Source: PLoS One. 2024 Feb 23;19(2):e0299028. doi: 10.1371/journal.pone.0299028 (PMC10889641; doi:10.1371/journal.pone.0299028)
Supplement: S1 Fig — (PDF) [file pone.0299028.s001.pdf]

Supplementary material

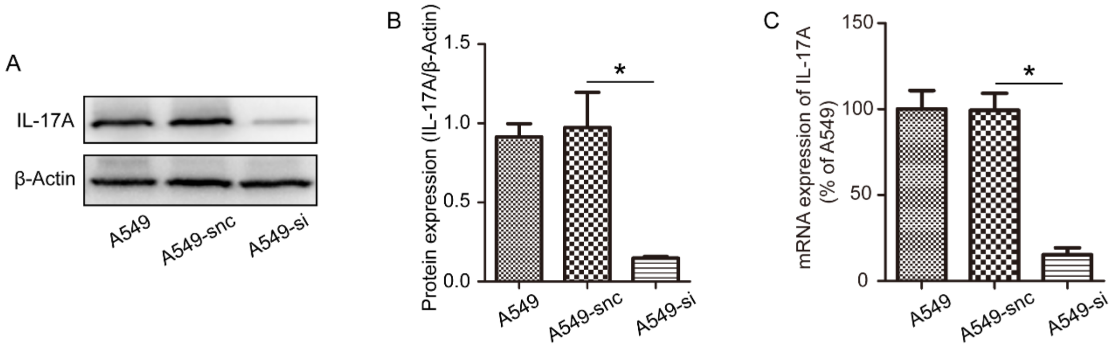

S1 Figure. Effects of siRNA-mediated IL-17A silencing in A549 cells. A specific siRNA targeting IL-17A mRNA was used to knockdown IL-17A expression. (A, B) IL-17A protein levels in cells were assessed via western blotting. Protein expression was normalized against β-Actin; untransfected cells (A549) served as a negative control. (C) *IL-17A* mRNA levels in siRNA-transfected and untransfected cells were quantified using real-time quantitative PCR. Data represent the mean ± SD of triplicate experiments. *P <0.05.
