# Supplementary material for: IL-17A deficiency inhibits lung cancer-induced osteoclastogenesis by promoting apoptosis of osteoclast precursor cells
Source: PLoS One. 2024 Feb 23;19(2):e0299028. doi: 10.1371/journal.pone.0299028 (PMC10889641; doi:10.1371/journal.pone.0299028)
Supplement: S2 Fig — (PDF) [file pone.0299028.s002.pdf]

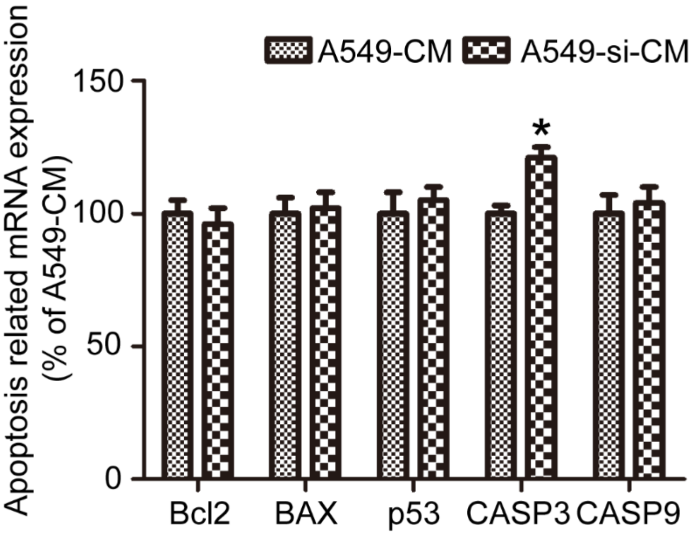

S2 Figure. Expression of apoptosis-related mRNA after treatment with A549-CM and A549-si-CM. mRNA expression of apoptosis-related genes was assessed in the presence of RANKL (50 ng/mL) after A549-CM and A549-si-CM treatment for 24 h. Data represent fold-changes in target gene expression normalized to that of *GAPDH* and are expressed as a percentage of the expression in cells treated with A549-CM, which was set to 100%. Data represent the mean ± SD (n = 4). *P <0.05.
