# Supplementary material for: IL-17A deficiency inhibits lung cancer-induced osteoclastogenesis by promoting apoptosis of osteoclast precursor cells
Source: PLoS One. 2024 Feb 23;19(2):e0299028. doi: 10.1371/journal.pone.0299028 (PMC10889641; doi:10.1371/journal.pone.0299028)
Supplement: S1 Table — (PDF) [file pone.0299028.s003.pdf]

S1 Table. Sequences of primers used in RT-PCR analysis

Gene	Primer sequence (5′–3′)
c-Fos	F: CGG GTT TCA ACG CCG ACT A
	R: TTG GCA CTA GAG ACG GAC AGA
NFATC-1	F: GGA GAG TCC GAG AAT CGA GAT
	R: TTG CAG CTA GGA AGT ACG TCT
TRAP	F: GCA GTA TCT TCA GGC GAG AAC
	R: TCC ATA GTG AAA CCG CAA GTA G
Catk	F: TAT GAC CAC TGC CTT CCA ATA C
	R: GCC GTG GCG TTA TAC ATA CA
Bax	F: GAT GCG TCC ACC AAG AA
	R: AGT AGA AGA GGG CAA CCA C
Bcl-2	F: TAC GAG TGG GAT GCT GG
	R: ACG ACG GTA GCG ACG AG
P53	F: TGG CCA TCT ACA AGC AGT CA
	R: GGT ACA GTC AGA GCC AAC CT
CASP3	F: GAA ACT CTT CAT CAT TCA GGC C
	R: GCG AGT GAG AAT GTG CAT AAA T
CASP9	F: AGT TCC CGG GTG CTG TCT AT
	R: GCC ATG GTC TTT CTG CTC AC
GAPDH	F: GGA GAA ACC TGC CAA GTA TGA
	R: TCC TCA GTG TAG CCC AAG A

F, forward; R, reverse.
