# Supplementary figures and images for: IL-17A deficiency inhibits lung cancer-induced osteoclastogenesis by promoting apoptosis of osteoclast precursor cells
Source: PLoS One. 2024 Feb 23;19(2):e0299028. doi: 10.1371/journal.pone.0299028 (PMC10889641; doi:10.1371/journal.pone.0299028)

FigS1

IL-17A

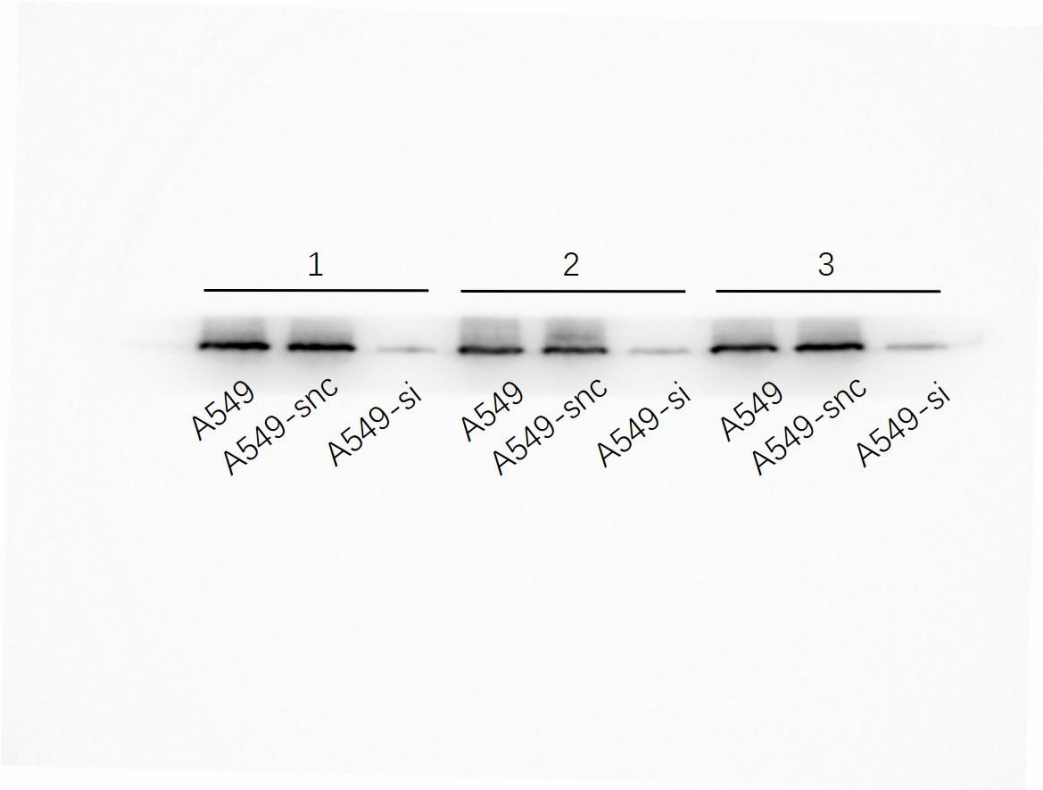

$\beta$ -Actin

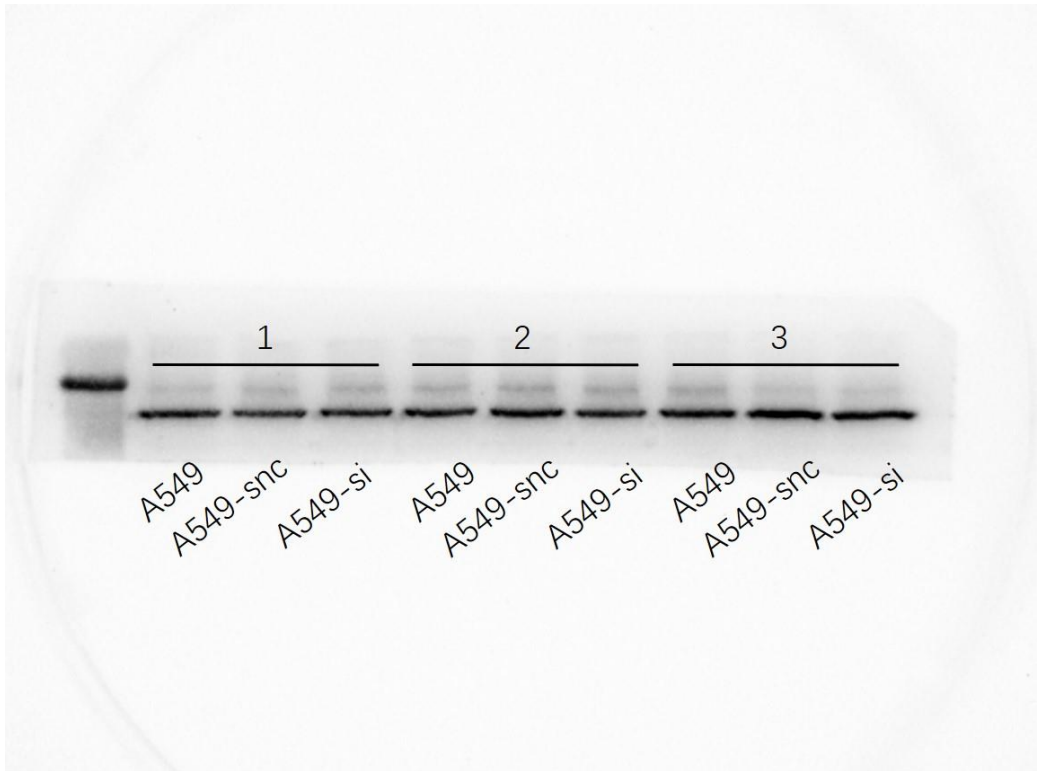

**Fig3**

Bcl2

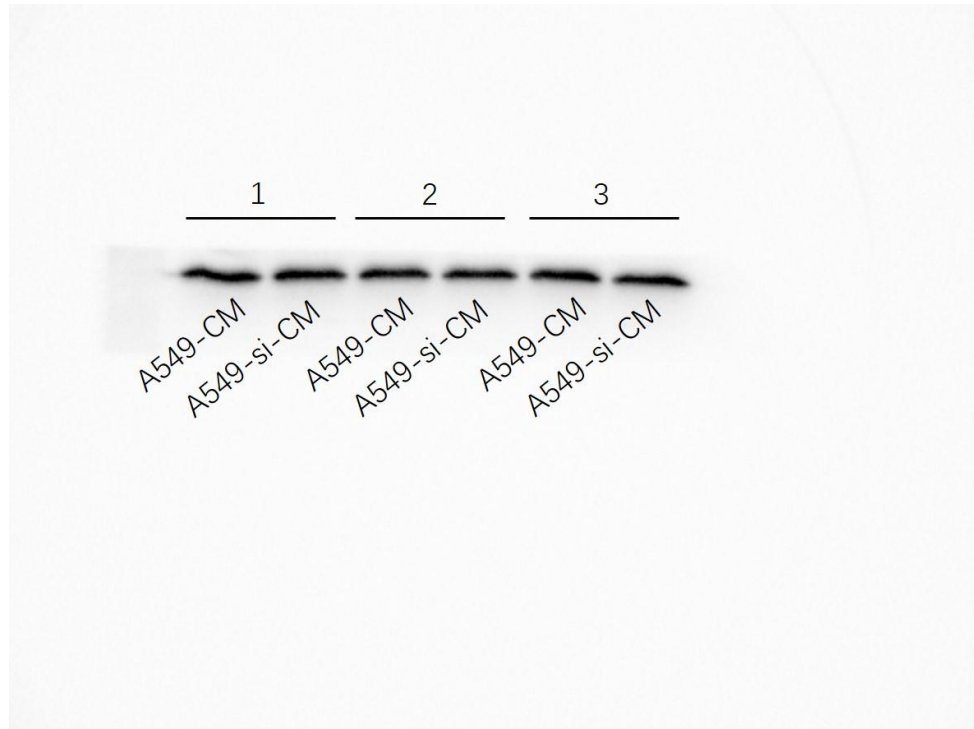

BAX

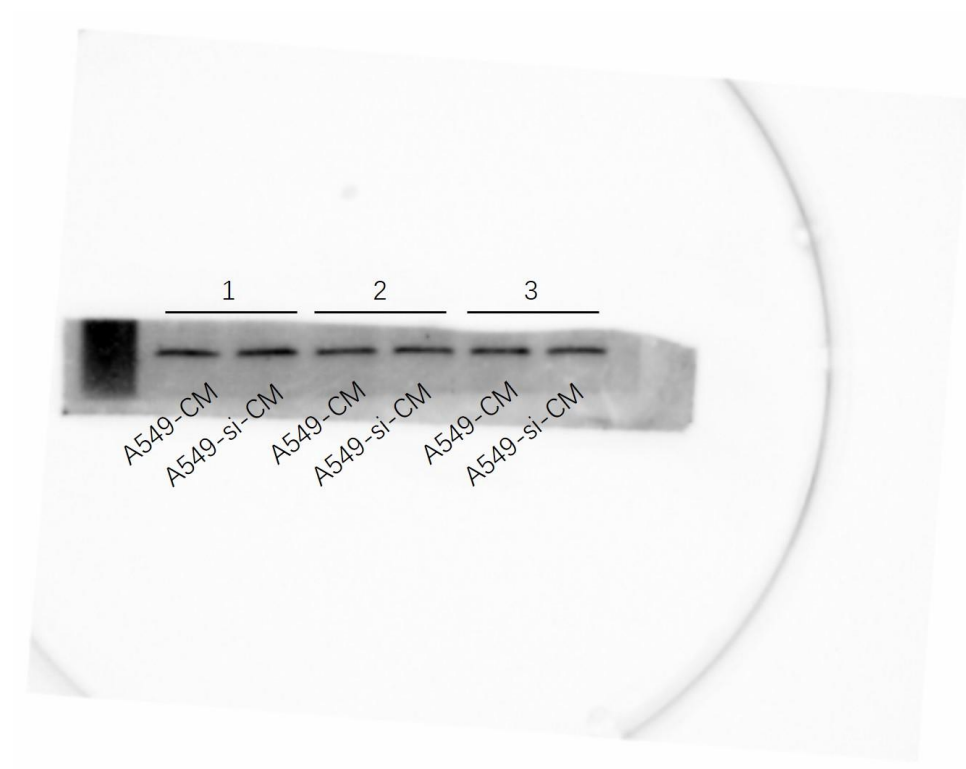

P53

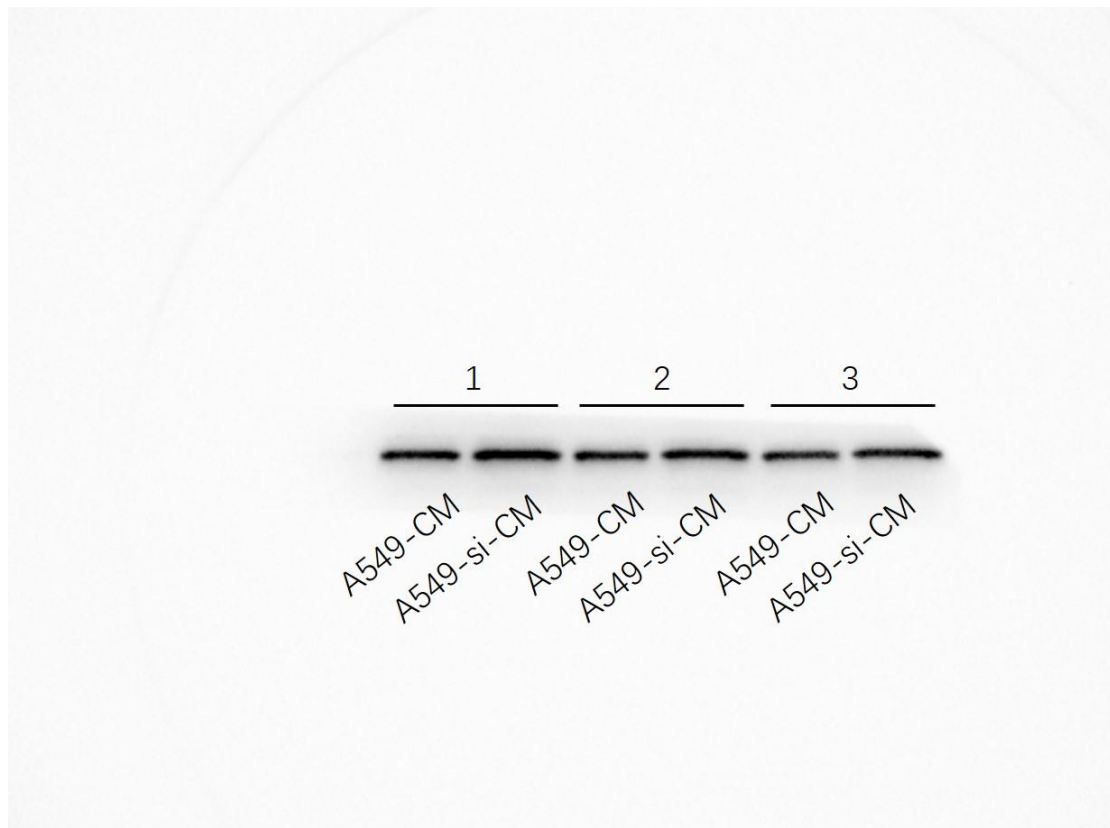

CASP3

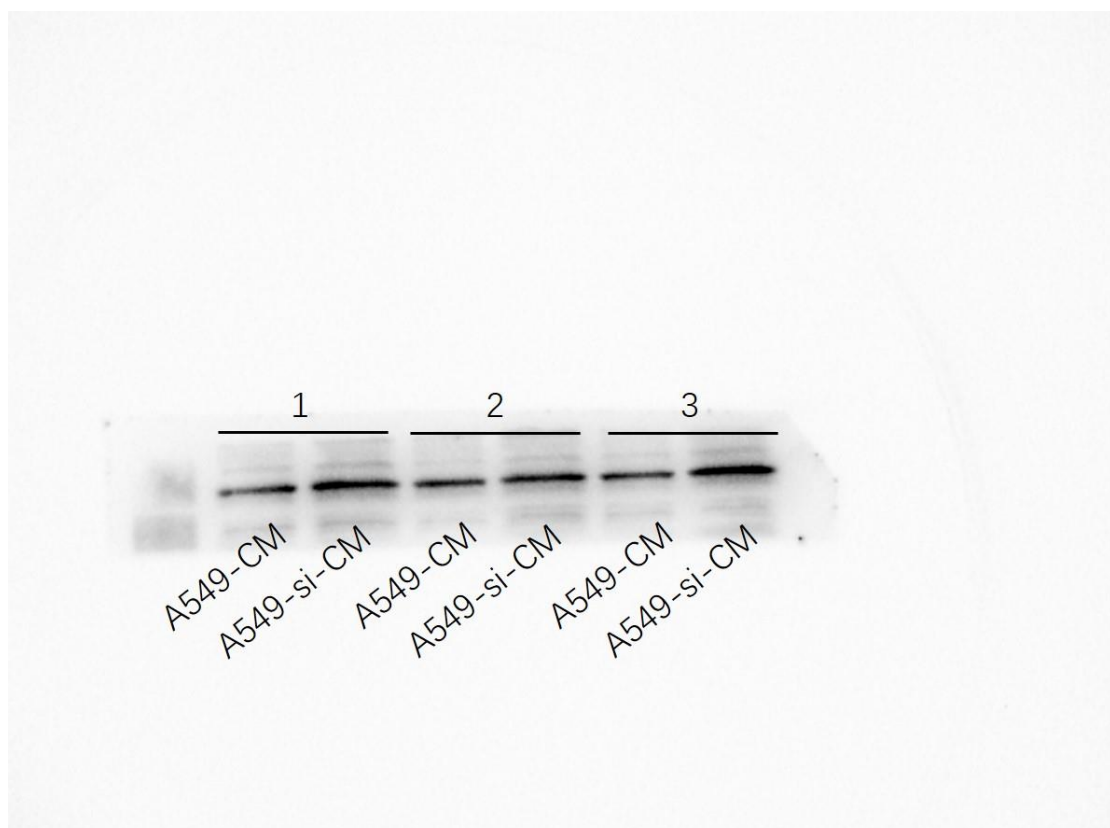

CASP9

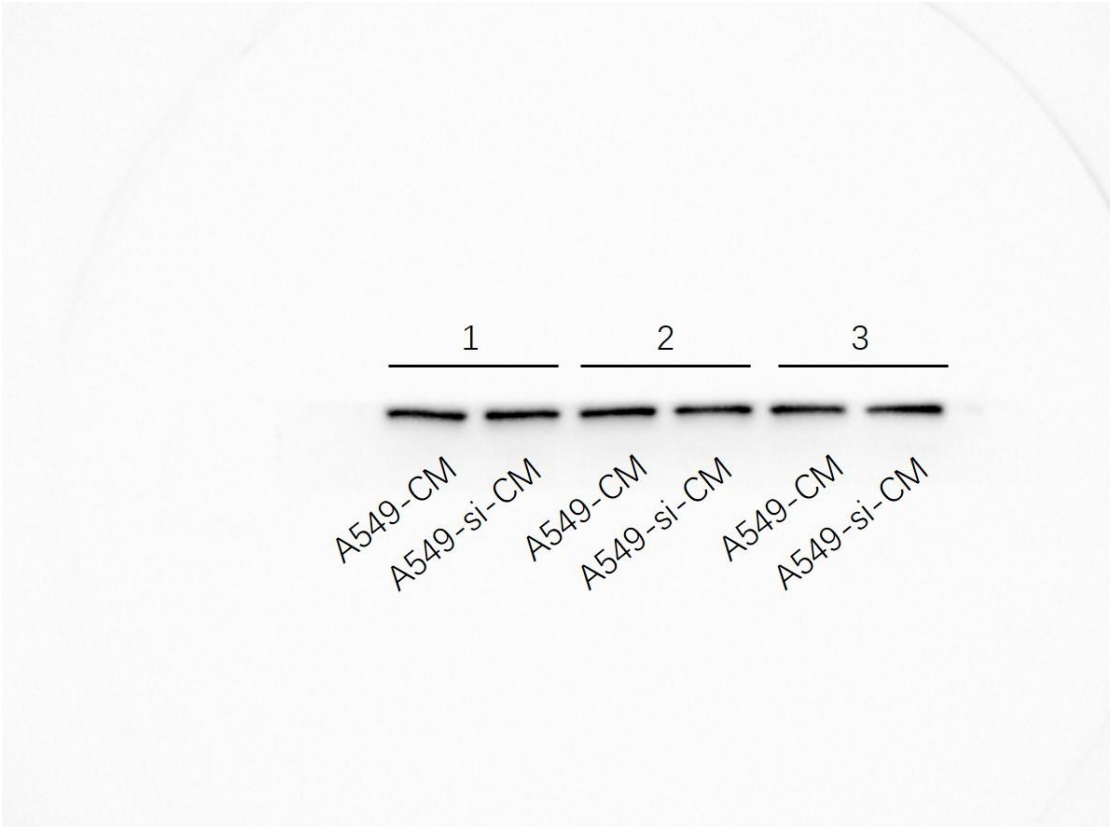

$\beta$ -Actin

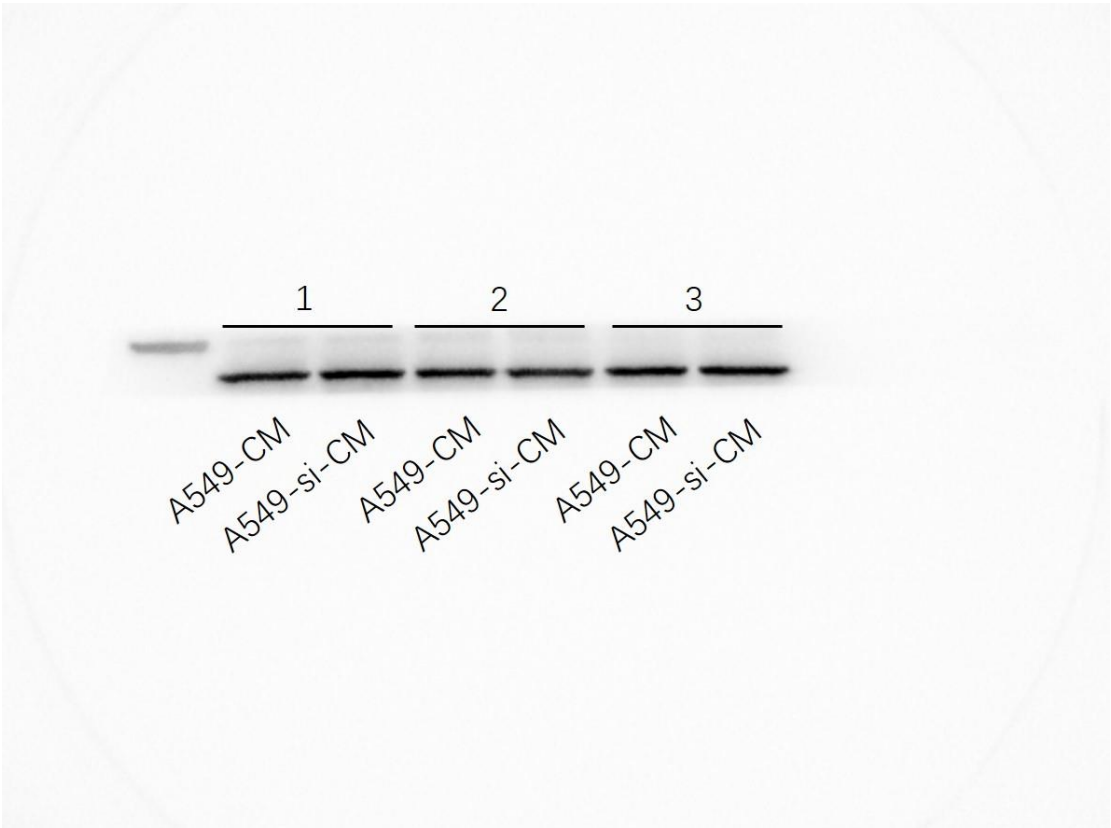

Supplement: S1 Raw images — (PDF) [file pone.0299028.s004.pdf]
